# TRPM Family Channels in Cancer

**DOI:** 10.3390/ph11020058

**Published:** 2018-06-07

**Authors:** Aline Hantute-Ghesquier, Aurélien Haustrate, Natalia Prevarskaya, V’yacheslav Lehen’kyi

**Affiliations:** 1 Laboratory of Cell Physiology, INSERM U1003, Laboratory of Excellence Ion Channels Science and Therapeutics, Department of Biology, Faculty of Science and Technologies, University of Lille, 59650 Villeneuve d’Ascq, France; ghesquier.aline@gmail.com (A.H.-G.); aurelien.haustrate@inserm.fr (A.H.); 2 FONDATION ARC, 9 rue Guy Môquet 94830 Villejuif, France

**Keywords:** TRPM channels, cancer, target, therapy

## Abstract

Members of the TRPM (“Melastatin”) family fall into the subclass of the TRP channels having varying permeability to Ca^2+^ and Mg^2+^, with three members of the TRPM family being chanzymes, which contain C-terminal enzyme domains. The role of different TRPM members has been shown in various cancers such as prostate cancer for mostly TRPM8 and TRPM2, breast cancer for mostly TRPM2 and TRPM7, and pancreatic cancer for TRPM2/7/8 channels. The role of TRPM5 channels has been shown in lung cancer, TRPM1 in melanoma, and TRPM4 channel in prostate cancer as well. Thus, the TRPM family of channels may represent an appealing target for the anticancer therapy.

## 1. Molecular Biology of TRPM Ion Channels Family

The transient receptor potential (TRP) superfamily of ion channels now consists of more than 30 cationic channels, most of which are permeable for Ca^2+^, and some also for Mg^2+^ [1]. This superfamily of TRP channels can be divided on the basis of sequence homology into seven main families, including the TRPC or canonical family, the TRPV or vanilloid family, the TRPM or Melastatin family, the TRPP or polycystin family, the TRPML or mucolipin family, the TRPA or ankyrin family, and the TRPN or NOMPC family [2].

It was suggested in the early 2000s that the TRP superfamily of channels possesses a molecular structure similar to that of voltage-gated potassium channels. Some homo- or hetero-tetrameric rearrangements were reported near the pore region, which are situated between the 5th and 6th hydrophobic transmembrane domains [1]. Our knowledge of the structure of TRP channels was substantially updated when the 3D structure was studied in detail for the TRPM2 [1,3], TRPM4 [1,4], and the TRPM8 channels [5,6].

TRP superfamily of channels plays an important role in non-excitable cells [7]. Moreover, it was suggested that many TRP-related proteins may also be expressed in the nervous system, and have important functions in sensory physiology [7]. 

There are four known subfamilies of TRPM channels: TRPM1/3, TRPM2/8, TRPM4/5, and TRPM6/7. TRPM channels exhibit differential permeability for Ca^2+^ and Mg^2+^, ranging from Ca^2+^- impermeable TRPM4/5 members to highly Ca^2+^ and Mg^2+^ permeable members such as TRPM6 and 7 [2]. Two members of the TRPM family have primary function in sensory perception, and three of them are chanzymes which contain C-terminal enzyme domains [8]. Actually, there are three TRPM members which, in their C-terminal regions, have enzymatic domains, such as TRPM2, TRPM6, and TRPM7 channels. Moreover, TRPM2-like channel-enzymes are widespread in eukaryotic species, where the pore-forming segments of invertebrate TRPM2-like proteins display high sequence similarity to those of Ca^2+^-selective TRPMs. Human TRPM2 is characterized by a loss of several conserved residues, and thus, selectivity to Ca^2+^ [9].

**Figure 1:**
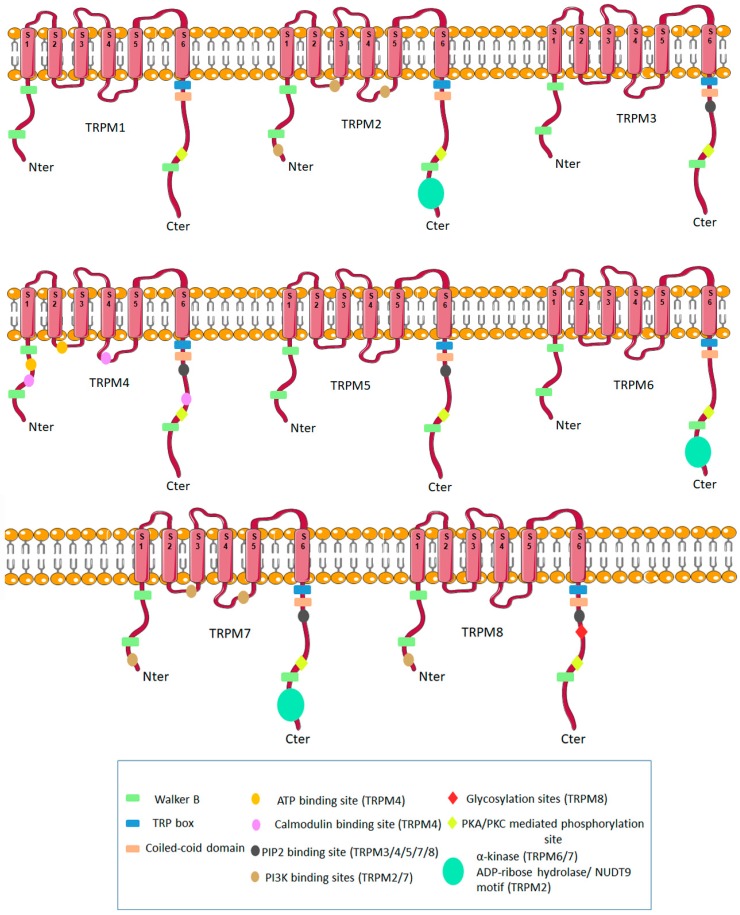
General structure of the TRPM family of channels.

The TRP box is situated in the C-terminal domain of the TRPM family, though there are no ankyrin repeats in the N-terminus (Figure 1). According to the latest data obtained using 3D cryostructure electronic microscopy, the TRP domain is not a continuous α-helix, but rather, has a break at about two-thirds of the helical structure length, thus, dividing the TRP domain into two segments [4]. The N-terminal part of all TRPM proteins is usually longer, by approximately 300–400 aa, than the same regions in TRPC and TRPV families. In this region, there is also a huge, approximately 700 aa TRPM-homology domain. The C-terminal sequences of the TRPM family display high variabilities, differing from as much as 1000 to 2000 aa.

There are three TRPM members which, in their C-terminal regions, have enzymatic domains, such as the TRPM2, TRPM6, and TRPM7 channels. The TRPM2 channel has an enzymatic domain similar to Nudix hydrolases, cleaving mono/di-nucleotidic polyphosphates [9]. This domain hydrolyses ADP-ribose into AMP and ribose-phosphate, though its efficiency is much lower than that of putative Nudix enzymes. On the other hand, the TRPM6 and TRPM7 channels contain kinase domains, which are classified as atypical alpha protein kinases [10].

The N-terminal domain with a TRPM homology region seems to be crucial for the self-assembly and trafficking of TRPM family channels. Moreover, it was shown that the splice variants of the TRPM1 and TRPM2 channels contain only the N-termini, without the C-termini and most or all of the transmembrane domains. This is enough to suppress the channel activity of the full-length isoform.

The first member of the TRPM family, TRPM1, was originally discovered as melastatin 1; as such, the other 8 members of this family were called TRPM for melastatin [8]. The gene of TRPM1 is comprised of 27 exons, with agene length of over 58 kb; it is situated on the chromosome 7 in mice. In humans, TRPM1 is localized on chromosome 15. The mRNA transcript of the TRPM1 gene spans approximately 5.4 kb [11,12]. TRPM1 encodes a 1603 aa protein of around 182 kDa, which is only expressed in the pigment cells of the skin and eye [11,12]. There is also a short N-terminal isoform of TRPM1 called MLSN1-S, which lacks all transmembrane domains yielding 500 aa protein [12]. It has been reported that this isoform and L form (MLSN1-L) are localized on the cell membrane, while the S form (MLSN1-S) is localized in the cytoplasm [13].

Like TRPM1, the TRPM3 gene yields avast number of different mRNA. Three transcripts of different lengths were detected in mouse brain [14], though Lee et al. [15] cloned six variants from human kidney. The presence of two, or maybe three alternative start positions, and two different C-terminal ends, allows a high variability of TRPM3 transcripts. The TRPM3 protein is expressed in the kidneys of miceand humans, andin human brain as well.

The differences seen in TRPM3 mRNAs transcripts, and therefore, in protein products, may result in different signaling mechanisms, such as store-depletion for the long variant, or induction by hypotonic solution for the short one [14,15]. This fact, in addition tothe specific expression of TRPM3 in kidney, suggest the involvement of this channel in renal osmo-homeostasis. On the other hand, the TRPM3 channel was characterized as the first cation channel activated by sphingosine detectable by TRPM3-mediated current in transfected HEK293 cells [16].

Intriguingly, TRPM3 channels, while forming non-selective but calcium-permeable membrane channels, may rapidly be activated by some steroids, such as pregnenolone sulphate, which makes them a sort of steroid receptor [17]. Moreover, the role of the TRPM3 ion channel was shown to be a heat sensor that acts independently of TRPV1 [18]. TRPM3^−/−^ mice were shown to be unable to avoid noxious heat, as well as to have a reduced avoidance of hot temperature zones [19].

TRPM6 and TRPM7 are the closest relatives of TRPM1 and TRPM3. Indeed, although their C-terminus domains are different, many amino acids are identical within their N-terminal sequences. Moreover, the transmembrane domains, the putative pore, and the small cytosolic domain following the sixth transmembrane domain show similarities amongthese four channels. TRPM6 and TRPM7 are characterized by a protein kinase domain at their C-termini [20,21]. The double activity—channel and enzymatic—of these two channels classify their as chanzymes or channel-kinases [20,22]. Disruption of TRPM6 kinase phosphorylation activity re-introduces Mg·ATP sensitivity to the heteromeric channel similar to that of TRPM7, which supports the notion that TRPM6 kinase plays a critical role in the control of the TRPM7/6 channel complex [23]. The ubiquitous TRPM7 protein is notably involved in magnesium homeostasis, just like TRPM6, with a fused alpha kinase domain [24].

This activity can be modulated by the intracellular magnesium concentration and by ATP. In contrastto TRPM7, TRPM6 has a specific expression in intestinal and renal epithelia [25]. However, a recent systematic assessment of TRPM6 using gene-modified mice revealed thecrucial role of TRPM6 in placental trophoblasts [26]. TRPM6 was also shown to specifically interact with its closest homolog, TRPM7 resulting in the assembly of functional TRPM6/TRPM7 complexes at the cell surface [27,28]. Theassociation of mTRPM6 with mTRPM7 allows high constitutive activity of mTRPM6/7 to occur in the presence of physiological levels of Mg^2+^ and Mg·ATP in epithelial cells. 

TRPM4 and TRPM5, calcium-activated sodium channels impermeable for calcium, mediate depolarization of the plasma membrane [29,30,31,32]. The selective expression of TRPM5 was initially found in the taste buds, which suggested that it may play a role in taste transduction [29,33]. Later, TRPM5 immunoreactivity was seen in other chemosensory organs, such as main olfactory epithelium and the vomeronasal organ, suggesting that TRPM5 is an intrinsic signaling component of mammalian chemosensory organs [34]. The putative binding sites for calmodulin (CaM), ATP, and proteinkinase C (PKC) phosphorylation sites within TRPM4 are consistent with its modulated activity by intracellular calcium, ATP, ADP, AMP [31] and the Ca^2+^-sensitivity of this channel. All putative ATP- binding sites are found in the N-terminus and the intracellular linker between the second and third transmembrane domain, together with the C-terminal CaM-binding sites and PKC phosphorylation sites.

TRPM2, the third chanzyme within the TRP family, demonstrates calcium activity coupled with cation channel activity coupled with an ADP-ribose pyrophosphatase activity through its enzymatic domain at the cytosolic C-termini of the protein [35,36,37]. TRPM2 is known to be activated by hydrogen peroxide involved in the host-defense system of the body [38], and constitutes a hydrogen peroxide-activated cation channel. Indeed, this channel is especially expressed in cells of the monocytic lineage, including various cultured macrophage cell lines, peripheral blood monocytes, and neutrophils [36,39,40], but also in the immune cells of the brain, the microglia [41]. TRPM2 can be found in other organs such as thepancreas, and in cell lines derived from pancreatic islet cells [42].

TRPM8, a protein of 1104 aa residue, displays the main conserved region characteristic of the TRP family. The gene of 102 kilobase pairs is localized on the chromosome 2, and contains 27 exons. The TRPM8 channel is a homotetramer formed by 130 kDa subunits. Several transcription factor binding sites on the promoter are known, such as NKX3-1, NKX2-5, USF1, MYC, LMOR, and ARNt. Alternative splicing of TRPM8 mRNA produces 11 isoforms. Within these splice variants, two of them—SM8α and SM8β—are specifically found in the prostate, and act as regulators of the full-length protein [5,43].

The C-terminal domain is crucial for the maturation, oligomerization, and trafficking of the channel to the plasma membrane [44,45]. Localized in the tail of this domain are the “TRP box”, a feature of TRP family members, and a binding site for PIP2. TRPM8 protein shows 8 putative glycosylation sites and an immunogenic epitope [46]. 

TRPM8 is expressed in several cells, but according to its role as molecular transducer of cold somatosensation in humans, TRPM8 channel shows its main expression in a subpopulation of primary afferent neurons from both the dorsal root and trigeminal ganglia, and in the nodose and geniculate ganglia in the peripheral nervous system. The protein has nevertheless also been found in others tissues like the prostate and genitourinary tract, bladder, vascular smooth muscle, liver, lung and odontoblasts [47]. In the cell, this channel shows a plasma membrane and a membrane rafts localisation. However, it has been shown that in prostate cancer cells, TRPM8 is also detected in endoplasmic reticulum membrane [47] and MAM [48]. Therecent discovery ofthe TRPM8 channel physical association with testosterone was published, and suggests that, in addition to a genomic role, testosterone plays a role in the direct regulation of the TRPM8 channel function [49]. Thus, the role of the TRPM8 channel may be suggested as being far beyond its well-established role in somatosensory neurons, and may also imply TRPM8 channel function in testosterone-dependent behavioral traits.

## 2. TRPM Channels in Cancer

### 2.1. Prostate Cancer

Most TRPM channels have been shown to be involved in prostate cancer. TRPM8 was originally identified by Tsavaler et al. [50] in 2001 by screening a prostate cDNA library. The TRPM8 gene was described as a novel prostate-specific gene owing to the fact that its expression increased over the transformation of prostate cancer. Indeed, the level of TRPM8 expression in normal prostate cells is very low, while in prostate cancer cells, it increases drastically. These results were confirmed by Fuessel et al. [51], who analyzed multiple tumor markers in primary prostate cancers. TRPM8 mRNA expression increases with the Gleason score and TNM stage. In androgen-dependent prostate carcinoma, TRPM8 seems to act as an inhibitor of the migration of prostate cancer cells by inactivating focal adhesion kinase in conjunction with partner proteins [52,53]. It was also revealed that TRPM8 activation by PSA reduced motility of the PC3 PCa cell line, suggesting that plasma membrane TRPM8 has a protective role in PCa progression [54]. Surprisingly, the overexpression of TRPM8 is not associated with changes in the level of androgen receptor (AR) mRNA expression. However, Henshall et al. [55] found that the expression of TRPM8 decreased markedly following anti-androgen therapy and when prostate cancer cells became androgen-independent, which supports the hypothesis that TRPM8 is regulated by androgens [56]. Indeed, the androgen dependence of TRPM8 expression is linked to the stage of differentiation of prostate epithelial cells [56,57].

The TRPM2 channel has been shown to play a role in prostate cancer particularly in prostate cancer cell proliferation. Indeed, the levels of TRPM2 mRNA are higher in prostate cancer tissue and in LnCaP and PC3 cell lines. A high expression of TRPM2 transcripts is found in 75% of malignant epithelial cells, compared to the matched benign cells of the surgical specimens. Contrary to benign cell lines, in whichTRPM2 is only expressed in the plasma membrane and the cytosol such as in lysosomes, PC3 cells show TRPM2 localization in their nuclei too. The role of TRPM2 in PCa cell proliferation is undeniable; it is shown by cell growth inhibition with the siRNA knockdown of TRPM2 via a mechanism which is independent of the activity of poly(ADP-ribose) polymerases of the channel [58,59].

Another TRPM channel identified as an actor in prostate cancer development is the TRPM4 channel. Actually, patients with prostate cancer display an elevation of gene expression of TRPM4. With a knockdown of TRPM4, a decrease of the migration, but not proliferation, of DU145 and PC3 cells was observed. This effect of TRPM4 has been shown to be induced by the regulation of SOCE in hPEC and DU145 cells [60]. On this basis, TRPM4 was identified as a cancer driver gene in androgen-insensitive prostate cancer through an increased migration.

TRPM7 is involved in the migration and invasion in PCa cells. In fact, this channel is up-regulated in PCa cells and tissues compared to prostate hyperplasia cells, and provokes an increased migration of these cells. In addition, TRPM7 deficiency by a knockdown in PCa cells suppressed migration and invasion of distinct PCa cell lines. Moreover, it also impacts the epithelial-mesenchymal transition (EMT) status by reversing it, while downregulating MMPs and upregulating E-cadherin [61].

### 2.2. Pancreatic Cancer

The main TRPM channel involved in pancreatic cancer is TRPM7. Indeed it is 13-fold overexpressed in cancer tissues compared to healthy ones, and its expression correlates with the PDAC progression. Importantly, TRPM7 expression is inversely related to patient survival. The mechanism by which TRPM7 modules BxPC-3 cell migration is Mg^2+^-dependent [62]. TRPM7 increases PDAC cell invasion through the regulation of the proteolytic axis, Hsp90α/uPA/MMP-2 pathway [63]. This axis is significantly decreased in TRPM7-deficient PDAC cells. 

TRPM2 is also negatively correlated with patient survival rate, compared with the control group. In fact, the moreTRPM2 is expressed in cancerous tissue, the shorter the survival times displayed by PDAC patients. The overexpression of TRPM2 has been shown to be associated with an increase incell proliferation and invasive ability. Therefore, the expression level of TRPM2 is noticeably linked with proliferation, invasive ability, and poor prognosis in patients with PDAC [64].

Finally, TRPM8 expression is also markedly up-regulated in human pancreatic adenocarcinoma cell lines and tissues, and is important for cellular proliferation. When TRPM8 is deficient in pancreatic cancer cells, a reduced ability of proliferation and cell cycle progression with elevated levels of cyclin-dependent kinase inhibitors is observed [65].

### 2.3. Lung Cancer

The TRPM7 channel is positively correlated with EGF expression, known as pro-oncogen. The depletion of TRPM7 in A549 lung cancer cells via siRNA inhibits cell migration [66].

TRPM8 contributes to an invasive phenotype in lung cancer via a synergic action with TRPA1. Indeed, results suggest that TRPM8 channels stimulate UCP2 to trigger metabolic transformation, whereas TRPA1 induces autophagy during adverse conditions, leading to an invasive phenotype [67].

The long noncoding RNA TRPM2-AS is widely up-regulated in non–small cell lung cancer tissues, compared with adjacent non-tumor tissues. This higher expression level of TRPM2-AS is correlated with higher TNM stages and larger tumor size. TRPM2-AS expression was inversely related to patient survival: patients with a high expression level of this TRPM2 variant had poorer survival rates than those with low TRPM2-AS levels. The knockdown of TRPM2-AS has also significantly inhibited cell proliferation [68].

The TRPM5 channel is likely to be involved in lung metastasis, as was shown by the increase in experimental lung metastasis when TRPM5 expression is enforced. TRPM5 activity increased the rate of acidic pH-induced MMP-9 expression, and required protein for the metastasis process. The use of triphenylphosphine oxide (TPPO), an inhibitor of TRPM5, in treatment of tumor-bearing mice, significantly reduced spontaneous lung metastasis [69].

### 2.4. Breast Cancer

TRPM7 and TRPM8 expression was shown to be correlated with breast cancer. Dhennin-Duthille et al. [70] observed high levels of TRPM7 and TRPM8 expression in human breast ductal adenocarcinoma (hBDA) tissue compared to adjacent non-tumor tissue. TRPM7 and TRPM8 expression is importantly correlated with the Scarff-Bloom-Richardson (SBR) grade, Ki67 proliferation index, and tumor size. TRPM7 impacts several processes in cancer development. The positive correlation between the Ki67 mitosis marker and the up-regulated TRPM7 channel in breast carcinoma tissue suggests the role of this channel in breast cancer cell proliferation. TRPM7 can also influence cell adhesion and migration via the regulation of myosin-IIA filament stability, and it influences protein localization by phosphorylating the heavy chain [22]. TRPM7-mediated migration and invasion of MDA-MB-435 breast cancer cells especially involved the mitogen-activated protein kinase (MAPK) signaling pathways. Silencing TRPM7 induces a significant reduction in the migration and invasion potential of MDA-MB-435 breast cancer cells, in addition to a decrease in the levels of phosphorylated Src and MAPK.

Concerning TRPM8 channels, they are highly expressed at both the mRNA and protein levels in the MCF-7 breast cancer cell line, as well as in breast adenocarcinomas, and are especially correlated with estrogen receptor positive (ER+) tumors [66].

TRPM2 is also overexpressed in situ and in invasive breast carcinoma, compared to normal breast tissue [71]. This channel likely acts as a protector of genomic DNA in breast cancer cells by minimizing DNA damage, thus promoting tumoral growth [72].

### 2.5. Melanoma

TRPM1 (also called melastatin) involvement in melanoma is undeniable. This channel was first discovered in the B-16 mouse melanoma cell line. TRPM1 expression steadily decreases during the progression of primary cutaneous and vertical growth phase melanomas. In fact, TRPM1 mRNA is almost or totally absent in around 80% of invasive primary melanomas. Thus, the TRPM1 gene is considered to bea tumor suppressor. In murine cell lines, Duncan et al. [73] showed that TRPM1 was expressed at high levels in poorly metastatic variants of the melanoma cell line, and expressed at very low levels in the highly metastatic melanoma cell line. In similar human melanoma experiments, Deeds et al. [74] found high levels of TRPM1 mRNA in melanocytic nevi, and noTRPM1 mRNA in melanoma metastases. In conclusion, the decreased expression of TRPM1 in melanoma development correlates with the melanoma cell transition from a low to a high metastatic phenotype, as well as with patient prognosis. For example, patients with stage I tumors, who display a diffuse expression of TRPM1, have an 8-year disease-free survival rate of 100%, while patients at the same stage but with no expression of TRPM1 have an 8-year disease-free survival rate of 77 ± 15%. The assessment is the same for patients with stage II disease with no TRPM1 expression: these patients have an 8-year disease-free survival rate that is significantly lower than that of patients at the same stage but who are expressing TRPM1. According to some studies, TRPM1 transcription regulation seems to occur via the binding of microphthalmia transcription factor (MITF), which is an essential transcription factor for the development of melanoma. Actually, depending on changes in MITF levels, TRPM1 mRNA expression is positively up- or down-regulated.

TRPM7 channels also act as protectors in both melanocyte physiology and in melanoma cells by acting as detoxifiers [66].

As for TRPM2, it is cited as a factor that can induce melanoma apoptosis and necrosis.

In normal cells, TRPM8 inhibits pigmentation of the melanocyte. However, in G-361 human melanoma cell line, menthol-mediated TRPM8 activity caused a prolonged increase in both the intracellular Ca^2+^ concentration and the amplitude of the current, drastically reduced the survival of melanoma cells. Therefore, TRPM8 channels play a role in melanoma proliferation [75].

### 2.6. Gastric Cancer

TRPM7 channels have been shown to be predominant actors in the growth and survival of human gastric adenocarcinoma cells. Kim et al. [76] noticed an abundant expression of TRPM7 messenger RNA and protein in AGS gastric cancer cells, the most commonly used line of human gastric adenocarcinoma cells. The blocking of TRPM7 channels with La^3+^ and 2-APB, or silencing TRPM7 expression with siRNA, inhibited the growth and survival of these cells. Kim et al. [76] later found that ginsenoside Rg3 inhibits the growth and survival of gastric cancer cells by blocking TRPM7 channel activity. 

TRPM2 regulates autophagy through a c-Jun N-terminal kinase (JNK)-dependent and mechanistic target of rapamycin-independent pathway. Indeed, the lack of the TRPM2 channel down-regulates the JNK-signaling pathway, and impairs autophagy, ultimately causing the accumulation of damaged mitochondria, and the death of gastric cancer cells. Thus, TRPM2 knockdown inhibits cell proliferation, and promotes apoptosis in gastric cancer cells [77].

### 2.7. Nasopharyngeal Carcinoma

Only TRPM7 has been reported as playing a role in nasopharyngaeal carcinoma. In fact, the impairment of TRPM7 channel function in NPC cells causes a significant reduction of cellular migratory potential. On the contrary, increased TRPM7 activity by TRPM7 activator (Bradykinin), and overexpression of the channel, promote migration in 5-8F and 6-10B cells. That is in favor of a pro-migratory role of TRPM7 in these cells. An extracellular Ca^2+^ chelator (EGTA), TRPM7 inhibitors (La^3+^ and 2-APB), as well as TRPM7 knockdown, significantly reduce the migratory potential of 5-8F and 6-10B cells [66]. Thus, TRPM7 channels enhance growth, but especially migration, by mediating Ca^2+^ influx in human head and neck carcinoma.

### 2.8. Others Cancers

Li et al. [78] showed that in human bladder cancer (BCa), TRPM8 was highly expressed in T24 cells. Moreover, activation of the channel by menthol could significantly decrease the viability of T24 cells. TRPM7 might be a potential actor in BCa tumorigenesis, by interfering BCa cell proliferation, motility, and apoptosis [79].

TRPM8 is also involved in osteosarcoma, by mediating proliferation and metastasis [80,81]. Indeed, Wang et al. [81] noticed that TRPM8 was overexpressed both in human osteosarcoma cells and osteochondroma specimens. In addition, TRPM8 knockdown causes a decrease in cell proliferation, migration, and invasion.

Just like in osteosarcoma, the TRPM8 channel is expressed at high levels, and acts as modulator of the invasion potential in Squamous Cell Cancer (SCC) [82,83].

Many studies established the presence of several TRP channels in liver cancer tissue, including TRPM4 and TRPM7 [66].

Numerous TRPM channels contribute to glioma invasion by inducing Ca^2+^ signaling, cytoskeleton changes, and migration; processes in which the TRPM family is known to be involved. Activation of TRPM8 channel by its agonist, menthol, increases the [Ca^2+^]_i_ in glioma cells, correlating with enhanced migration. Cell shape, adhesion and migration are regulated by actomyosin contractility. Moreover, TRPM7 has been found to link receptor-mediated signals to actomyosin remodeling and cell adhesion in HEK293 cell lines. Bradykin mediated TRPM7 activation causesincreased Ca^2+^ signal and kinase-dependent interaction with the actomyosin cytoskeleton. The overexpression of TRPM7 resulted in cell spreading, adhesion, and formation of focal adhesions, by increasing the intracellular Ca^2+^ levels. In addition, the Ca^2+^ permeable TRPM2 channel enhanced cell death induced by H_2_O_2_ was shown in human A172 glioma cells [84].

Finally, human cervical-uterine tumor samples and cervical-uterine cancer derived cell lines display a high TRPM4 mRNA level and an amplification of TRPM4 channel. A reduced TRPM4 expression decreases proliferation of HeLa cervical cancer-derived cells. The constitutive silencing of this channel in these cells induces GSK-3b dependent degradation of β-catenin and reduced β-catenin/Tcf/Lef-dependent transcription, while the overexpression of TRPM4 in T-REx 293 cells increased cell proliferation and β-catenin levels. Thus, TRPM4 channels enhance the proliferation of T-REx 293 and HeLa cervical-uterine cancer cells by promoting nucleus stability and the function of catenin as a transcriptional co-factor. For this reason, TRPM4 is identified as a key actor in cervix cancer development via the modulation of the β-catenin pathway, whose impairment is frequently associated with this cancer [84].

## 3. Conclusions

**Figure 2:**
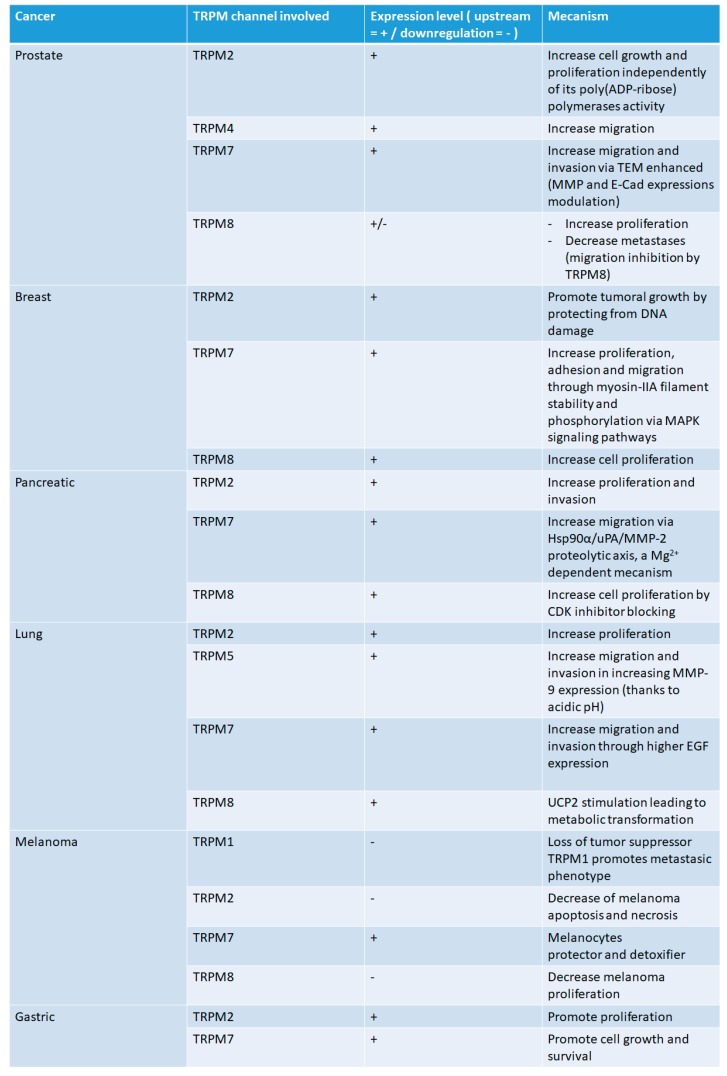
Role of different TRPM channels in cancer. (+) for the increase of function, (−) for the decrease of function.

TRPM channels are largely represented in many human pathologies included cancer. Having variable permeability to Ca^2+^ and Mg^2+^ makes them non-selective cationic channels, while three of them, TRPM2/6/7, are chanzymes, a feature which is presently unknown in ion channels. The role of different TRPM members has been shown in various cancers, such as prostate, breast, pancreatic, lung, and melanoma, and is summarized in Figure 2.
